# Assessment of Peripheral Platelet to Lymphocyte Ratio and Prognostic Nutritional Index in the Efficacy and Prognosis of Radiotherapy for Cervical Cancer

**DOI:** 10.3390/curroncol30030216

**Published:** 2023-02-27

**Authors:** Zhongrong Gao, Mengli Zhao, Xiaojing Yang, Jie Fu

**Affiliations:** Department of Radiotherapy, Shanghai Sixth People’s Hospital Affiliated to Shanghai Jiao Tong University School of Medicine, Shanghai 200233, China

**Keywords:** cervical cancer, prognosis of radiotherapy, prognostic nutritional index, platelet to lymphocyte ratio

## Abstract

This study aimed to evaluate the correlation between the pre-treatment peripheral platelet-to-lymphocyte ratio (PLR) and the prognostic nutritional index (PNI) with the efficacy and prognosis of radiotherapy for cervical cancer. A total of 110 patients with cervical cancer who received radiotherapy at our hospital from November 2017 to November 2020 were retrospectively analysed. The cut-off values of PLR and PNI were obtained using the receive operating characteristic curve (ROC) and the Youden index. The patients were divided into high PLR and low PLR and high PNI and low PNI groups. We compared the clinical characteristics, 3-year overall survival (OS), and progression-free survival (PFS) between the high and low PLR groups, as well as the high and low PNI groups of patients. Cox regression was used to analyse the factors influencing OS and PFS. The median follow-up duration was 26 months. The optimal cut-off value for PLR was 186.88 and that for PNI was 47.35. The 3-year OS values were 81.00% and 97.10% for the high PLR (PLR > 186.88) and low PLR (PLR ≤ 186.88) groups, respectively, and the 3-year PFS values were 59.50% and 88.20% for the high PLR and low PLR groups, respectively, with statistically significant differences (*p* < 0.05). The 3-year OS values were 97.50% and 74.20% for the high PNI (PNI > 47.35) and the low PNI (PNI ≤ 47.35) groups, respectively, and the 3-year PFS values were 87.30% and 51.60% for the high PNI and low PNI groups, respectively, with statistically significant differences (*p* < 0.05). Multifactorial Cox regression analyses revealed that high PLR value (PLR > 187.88), low PNI value (PNI ≤ 47.35), histological type, and FIGO stage were independent risk factors for the OS of cervical cancer. Pretreatment PNI values and PLR values can be used as simple and feasible predictors of clinical efficacy and prognosis for patients treated with radiotherapy for cervical cancer.

## 1. Introduction

Cervical cancer is a malignant tumour that seriously threatens the life and health of women. Cervical cancer has the second highest occurrence rate (second to breast cancer) among female malignant tumours [1]. Women aged 40–60 years are more likely to develop cervical cancer, although there has been trend towards younger ages [2]. Currently, surgery is one of the primary methods for treating cervical cancer; however, the postoperative overall survival (OS) rate remains unsatisfactory, and postoperative treatment is mostly supplemented with radiotherapy and chemotherapy to prolong patient survival [3]. However, disease recurrence and progression result in the death of approximately one-third of the patients each year [4]. Therefore, exploring the biological mechanisms of cervical cancer progression and the influencing factors of prognosis is essential. Recent studies have demonstrated that cervical cancer is closely associated with the body’s inflammatory response and nutritional status [5,6]. The platelet-to-lymphocyte ratio (PLR) and the prognostic nutritional index (PNI) are valid indicators that reflect the systemic inflammatory response and nutritional status [7,8]. Reportedly, PLR and PNI affect the treatment efficacy and prognosis of various malignancies, such as ovarian cancer, gastric cancer, oesophageal cancer, and breast cancer [9,10,11,12]. The higher the PLR and the lower the PNI, the higher the risk of malignant tumour progression and metastasis, thus resulting in poor patient prognosis. Age, tumour size, clinical stage, and lymph node metastasis are factors affecting cervical cancer prognosis [13,14]. Few reports exist on the relationship between PLR, PNI, and the prognosis of patients with cervical cancer who received radiotherapy. Herein, we analysed the potential applicability of PLR and PNI in the prognostic evaluation of cervical cancer by comparing the clinical characteristics, efficacy, and survival differences of patients with cervical cancer in various pre-treatment PLR and PNI groups.

## 2. Materials and Methods

### 2.1. General Information

A total of 110 women with histologically confirmed cervical cancer (the International Federation of Gynaecology and Obstetrics (FIGO), stages I–IV) from our institution were included in this study. Each patient was routinely examined at baseline, and the PLR and PNI were calculated based on blood test results. The inclusion criteria were as follows: (1) pathologically confirmed cervical cancer before treatment; (2) the exclusion of other pelvic diseases; (3) patients with normal liver and kidney function (blood urea nitrogen ≤ 25 mg/dL, creatinine ≤ 1.5 mg/dL, and bilirubin ≤ 2 mg/dL); (4) patients with a history of other tumours before treatment; and (5) patients with complete haematological data before radiotherapy initiation. The exclusion criteria were as follows: (1) combined or secondary dichotomous carcinoma; (2) cervical cancer accompanied by an acute injury or acute and chronic inflammatory response; (3) patients with distant metastasis; (4) patients with blood-related diseases (e.g., aplastic anaemia and leukaemia); (5) patients with incomplete or missing follow-up data; (6) patients who were hypersensitive to drugs or devices used in treatment; (7) cervical cancer combined with the organic lesions of vital organs, such as the liver and kidney; (8) pregnant or breastfeeding women; (9) cervical cancer combined with serious infectious diseases; and (10) patients with immune dysfunction (such as human immunodeficiency virus infection) or immune hyperfunction (such as autoimmune disease).

### 2.2. Clinical Data

Patient clinical data include age, the FIGO stage, histological type, lymph node metastasis, tumour diameter, height, weight, and haematological data (lymphocyte, albumin, and platelet levels) before radiotherapy initiation. The PNI was determined as follows: PNI [12] = 5 × lymphocyte count (×10^9^/L) + serum albumin (g/L). The PLR was determined as follows: PLR [15] = platelet/lymphocyte ratio.

### 2.3. Method

#### 2.3.1. Treatment Methods

All patients received external pelvic radiotherapy at a total dose of 45.0–50.4 Gy (1.8–2.0 Gy/fraction, 5 fractions/week, 25–28 fractions in total). Some patients received brachytherapy.

#### 2.3.2. Therapeutic Evaluation

The tumours were clinically evaluated by direct observation or by measuring the size of the tumour lesion, and some cases were evaluated with reference to a vaginal Bright scan ultrasound or computed tomography measurements. According to the World Health Organisation (WHO) criteria for the recent evaluation of solid tumours, complete remission (CR) is defined as the complete disappearance of tumour lesions; partial remission (PR) is defined as a reduction of more than 50% of the maximum diameter of the tumour lesions; stable disease (SD) is defined as a reduction of less than 50% of the maximum diameter of the tumour lesions or an increase of no more than 25%; and progressive disease (PD) is defined as an increase of more than 25% of the maximum diameter of tumour lesions or the appearance of new lesions. CR and PR were considered clinically effective (CR + PR), and SD and PD were considered clinically ineffective (SD + PD).

#### 2.3.3. Follow Up

The final follow-up was conducted in May 2022, and the follow-up methods included outpatient review, inpatient review, telephonic follow-up, or text message follow-up. All patients were reviewed every 3 months for the first 2 years after treatment and every 6 months after 2 years. The follow-up examination comprised the following: gynaecological physical examination, blood test, pelvic magnetic resonance imaging, chest radiography, etc. Progression-free survival (PFS) was defined as the time from the patient’s definitive diagnosis to disease recurrence or metastasis. OS was defined as the time from the patient’s definitive diagnosis to death or the last follow-up visit.

### 2.4. Statistical Analysis

Statistical analysis was performed using Statistical Package for the Social Sciences (SPSS) 24.0 statistical software. Chi-square tests were used to compare groups, and data were expressed as rates (%). The optimal cut-off values for PLR and PNI were calculated using receiver operating characteristic (ROC) curves. Survival analysis was performed using the Kaplan–Meier method and the log-rank test, wherein the survival curves of the patients’ OS and PFS were plotted and between-group data differences were compared. The Cox regression model was used to analyse the factors affecting patient prognosis through univariate and multivariate analyses. Statistical significance was set at *p* < 0.05.

## 3. Results

### 3.1. Basic Patient Outcomes and Prognosis

Concurrent radiotherapy was administered to all patients. Among them, 73 were treated effectively (CR + PR) and 37 were not (SD + PD), according to WHO criteria. All patients completed follow-up with a median follow-up duration of 26 months. All patients had a 3-year OS rate of 90.91% (Table 1).

### 3.2. Predictive Value of PLR and PNI on the Survival of Patients with Cervical Cancer

**Figure 1:**
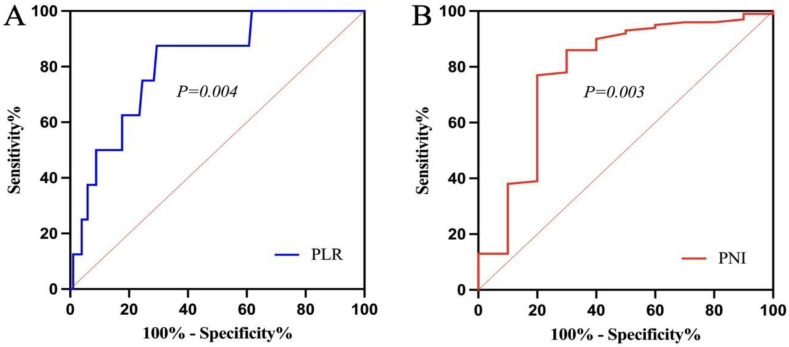
Receiver operating characteristic curve ((**A**) platelet-to-lymphocyte ratio; (**B**) prognostic nutritional index).

Pre-treatment PLR and PNI were used as test variables, and the survival outcome was used as the state variable while plotting the ROC curves (Figure 1A,B). According to the ROC curves, the sensitivity and specificity of PLR were 0.875 and 0.706, respectively, and the Youden index was 0.581. The corresponding optimal cut-off value at this time was 186.88. The patients were categorised into the high PLR group (PLR > 186.88) and the low PLR group (PLR ≤ 186.88). Similarly, the optimal cut-off value of PNI was 47.35, based on which the patients were categorised into the high PNI group (PNI > 47.35) and the low PNI group (PNI ≤ 47.35).

### 3.3. Correlation Analysis of PLR, PNI, and Clinical Characteristics of Patients with Cervical Cancer

Statistically significant differences were observed in terms of the histological type, lymph node metastasis, and prognosis between the high PLR and the low PLR (*p* < 0.05) groups. No significant difference was observed in terms of age, the FIGO stage, and tumour diameter between the two groups (*p* > 0.05) (Table 2).

Statistically significant differences were observed in terms of the age and prognosis between the high PNI and the low PNI (*p* < 0.05) groups, and no significant differences were observed in terms of the FIGO stage, histological type, lymph node metastasis, and tumour diameter between the two groups (*p* > 0.05) (Table 3).

### 3.4. Survival Analysis

**Figure 2:**
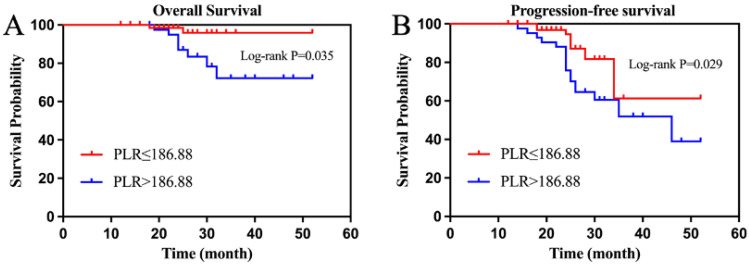
(Platelet-to-lymphocyte ratio) survival curve ((**A**) overall survival; (**B**) progression-free survival).

**Figure 3:**
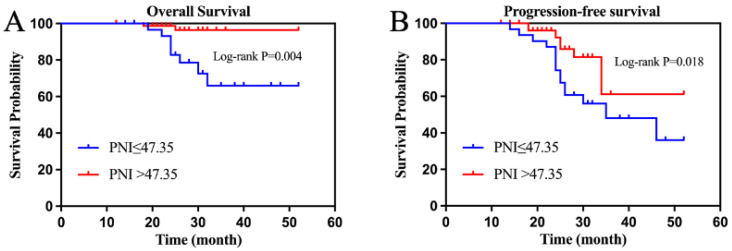
(Prognostic nutritional index) survival curve ((**A**) overall survival; (**B**) progression-free survival).

By the final follow-up, the median OS and PFS had not been achieved in the entire group, whose 3-year OS and PFS were 90.91% and 65.50%, respectively. The 3-year OS was 81.00% and 97.10% (*p* = 0.035), and the 3-year PFS was 59.50% and 88.20% (*p* = 0.029) in the high PLR and low PLR groups (Figure 2A,B). The 3-year OS was 97.50% and 74.20% (*p* = 0.004) and the 3-year PFS was 87.30% and 51.60% (*p* = 0.018) in the high and low PNI groups, respectively (Figure 3A,B).

### 3.5. Univariate Cox Analysis of PLR and PNI

Based on the Cox univariate analysis results, the risk factors for OS were as follows: the FIGO stage (III + IV), histological type (non-SCC), pre-treatment PLR > 186.88, and PNI ≤ 47.35 (Table 4). The risk factors for PFS were as follows: age (≥50 years), histological type (non-SCC), PLR >186.88, and PNI ≤ 47.35 (Table 5).

### 3.6. Cox Regression Analysis of Cervical Cancer Prognosis

The PNI ≤ 47.35, PLR > 186.88, histological type (non-SCC), and FIGO stage (III and IV) were independent poor prognostic factors affecting OS in patients with cervical cancer, based on the Cox multifactorial regression analysis (Table 6). The histological type (non-SCC) and age ≥ 50 years were independent prognostic risk factors affecting PFS (Table 7).

## 4. Discussion

It has been reported that over 80% of the newly diagnosed cases of cervical cancer occur in developing countries each year, where the disease is characterized by high incidence and mortality rates [16]. This significantly increases the economic burden on patients’ families and society. Therefore, it is of great importance to effectively predict prognosis and provide personalized interventions to improve patient outcomes and enhance their quality of life [17]. Currently, factors such as the cancer stage, lymph node metastasis, pathological type, and degree of tumour differentiation are clinically used to evaluate the prognosis of patients [18]. However, except for a few factors, such as staging, that can be evaluated before treatment, most of the other factors can only be evaluated after treatment. In addition, the ribonucleic acid and human papillomavirus expressions have also been demonstrated to serve as factors for the prognostic assessment of patients with cervical cancer [10,19]; however, their clinical applicability is limited because they are expensive and challenging to detect. The prognosis of patients depends on discovering a predictive indicator that can be assessed prior to radiotherapy and is convenient to detect. The study of tumour development, invasion, and metastasis in relation to genomics and proteomics has continued to advance in recent years [20]. Researchers have gradually discovered that the body’s nutritional status, the level of the systemic inflammatory response, and the immune regulatory mechanisms might affect the processes of tumour development, invasion, and metastasis to some extent. Clinical attention to PNI and PLR has been growing as a result of ongoing research on immune-nutritional indicators of malignant tumours.

Onodera et al. [21] first introduced the PNI. Serological albumin levels and lymphocyte counts, both of which use indicators that indirectly reflect the nutritional status and the immune performance of the patient to some extent, are used to evaluate this index [12]. The PNI is an objective and simple assessment tool that is easily accessible non-invasive. Serum albumin is one of the most important clinical indicators for assessing a patient’s nutritional status. Several studies have demonstrated that low serum albumin levels are associated with a poor prognosis in patients with various cancers [22]. Graziano et al. [23] retrospectively analysed the serum albumin levels of 2425 patients with non-metastatic invasive breast cancer (stages I–III) and reported that lower serum albumin levels served as a prognostic factor for poor survival in patients with early stage breast cancer, regardless of their stage. When patients with cervical cancer are malnourished, the postoperative recovery is poor, the body’s immunity and resistance are affected by varying degrees, the treatment tolerance is poor, and the risk of postoperative recurrence or disease progression is high, resulting in poor prognosis. Ida et al. [24] found that pre-treatment low PNI values were significantly associated with poorer OS in cervical cancer patient compared to those with high PNI values. Moreover, low PNI values were indicative of malnutrition in recurrent cervical cancer patients. Lymphocytes play a crucial role in the body’s immune response function, which can reflect the body’s immune, nutritional, and inflammatory response status [20,25]. When lymphocyte levels are normal, cytotoxic lymphocyte proliferation activation can effectively inhibit malignant tumour cell proliferation or migration and prevent cervical cancer progression or recurrence, thereby improving patient prognosis. Therefore, PNI can reflect a patient’s nutritional status and immune function, and its role in assessing the efficacy and prognosis has gained increasing attention. Xiao et al. [26] retrospectively analysed 193 patients with oesophageal cancer who received radical radiotherapy and demonstrated that the OS rate was higher in the high PNI group than in the low PNI group. Haraga et al. [27] reported that reduced PNI values were an independent poor prognostic factor for a patient’s OS and PFS through a study of patients with cervical cancer who received synchronous radiotherapy.

Non-specific inflammatory responses play an extremely important role in the progression of tumours. This may be because inflammatory factors release large amounts of reactive oxygen species and proteases, causing DNA oxidative damage in cells, ultimately triggering tumours [28]. PLR is a common haematological indicator reflecting the systemic inflammatory response obtained from the platelet-to-lymphocyte ratio [29]. Platelets are part of the inflammatory response, with reactive thrombocytosis being more common in solid tumours. Platelets can release various factors, such as platelet-derived growth factor (PDGF), vascular endothelial growth factor (VEGF), and transforming growth factor beta (TGF-β). These factors can promote the proliferation and adhesion of malignant tumour cells, thus affecting tumour growth and metastasis [30]. Thrombocytosis and lymphopenia are associated with the host’s degree of systemic inflammatory response. An elevated PLR indicates a relative increase in the platelet count or a relative decrease in the lymphocyte count. Several studies have demonstrated that PLR can be used not only as an indicator for the evaluation of immune function status but also as a poor prognostic factor for various malignant tumours [31].A retrospective study of 389 patients with advanced non-small cell lung cancer reported that a PLR below the cut-off value was associated with a longer PFS (*p* = 0.028) and OS (*p* = 0.001) and a higher objective response rate (*p* = 0.04) [32]. Raungkaewmanee et al. [9] demonstrated that pre-treatment PLR was associated with prognosis in epithelial ovarian cancer, with a lower survival rate and PFS observed in the high PLR group. Ma et al. [33] performed a meta-analysis of 3668 patients with cervical cancer and demonstrated that elevated pre-treatment PLR was associated with poorer OS and could be used as a biological marker for poor prognosis in patients with cervical cancer.

Herein, we retrospectively analysed 110 patients with cervical cancer who received radiotherapy and were divided into the high PLR, low PLR, high PNI, and low PNI groups. Significant differences were observed in terms of the age and prognosis between the high PNI and low PNI groups (*p* < 0.05), while significant differences were observed in terms of the histological type, lymph node metastasis, and prognosis between the high PLR and low PLR groups (*p* < 0.05). This reflects the role of PNI and PLR in tumour formation and suggests a link between PLR and PNI and tumour progression. When the relationship between PNI, PLR, and prognosis was further explored, we observed that the 3-year OS and PFS were significantly higher in the high PNI group than in the low PNI group (*p* < 0.05), and 3-year OS and PFS were significantly higher in the low PLR group than in the high PLR group (*p* < 0.05). PNI and PLR were found to be independent influencing factors on OS based on multifactorial Cox regression analysis (*p* < 0.05). However, PNI and PLR were not independent influencing factors for PFS (*p* > 0.05). This indicates that higher pre-treatment PNI values or lower pre-treatment PLR values were associated with better OS for patients. Nevertheless, the predictive value of PNI and PLR on PFS requires further study.

However, our study has certain limitations. First, the sample size of this study was small, and its retrospective single-centre design might have introduced some potential bias. Second, the patient survival varied widely, ranging from 1 to 4 years after diagnosis. Therefore, the survival analysis results might be biased. Third, the serial PNI or PLR counts for each patient during concurrent radiotherapy or radiotherapy alone were lacking. As a dynamic marker, the continuous measurement of PNI or PLR during treatment can potentially help in the early identification of patients who will not benefit from a single treatment. Changes in the PLR need to be further evaluated by clinical trials wherein the data can be prospectively analysed. Fourth, there is still no consensus on the optimal PNI and PLR cut-off values. Some studies used the median value to define the cut-off value, whereas some studies used the ROC curve. Our findings require more scientific evidence, which should come from prospective multicentre trials with larger sample sizes.

In summary, preoperative peripheral blood PNI and PLR could serve as predictive indicators for evaluating the curative effect and prognosis of patients with cervical cancer. Low PNI and high PLR indicate poor prognosis. At the same time, both indicators are easy to detect and will not increase the economic burden on patients. These indicators are expected to serve as clinical parameters for predicting the efficacy and prognosis of radiotherapy in patients with early stage cervical cancer, thus guiding clinical decision making.

## Figures and Tables

**Table 1:** Basic patient characteristics and efficacy.

Characteristic	Number
Age (year)	
<50 year	61 (55.50%)
≥50 year	49 (44.50%)
FIGO stage	
I	14 (12.70%)
II	38 (34.50%)
III	53 (48.30%)
IV	5 (4.50%)
Histological type	
SCC	97 (88.20%)
Non-SCC	13 (11.80%)
Lymph node metastasis	
Negative	57 (51.80%)
Positive	53 (48.20%)
Maximum tumour size (cm)	
>4 cm	65 (59.10%)
≤4 cm	45 (40.90%)
Prognosis	
Effective	73 (66.40%)
Ineffective	37 (33.60%)

FIGO, International Federation of Gynaecology and Obstetrics; SCC, squamous cell carcinoma.

**Table 2:** Association of PLR with the clinical characteristics of patients with cervical cancer.

Variable	PLR (High)	PLR (Low)	X^2^	P
Age (year)				
<50 year	22 (52.40%)	39 (57.40%)	0.26	0.61
≥50 year	20 (47.60%)	29 (42.60%)		
FIGO stage				
I + II	16 (38.10%)	36 (52.90%)	5.51	0.169
III + IV	26 (61.90%)	32 (47.10%)		
Histological type				
SCC	33 (78.60%)	64 (94.10%)	6.02	0.014
Non-SCC	9 (21.40%)	4 (5.90%)		
Lymph node metastasis				
Negative	16 (38.10%)	41 (60.30%)	4.27	0.039
Positive	26 (61.90%)	27 (39.70%)		
Maximum tumour size (cm)				
>4 cm	25 (59.50%)	40 (58.80%)	0.05	0.94
≤4 cm	17 (40.50%)	28 (41.20%)		
Prognosis				
Effective	20 (47.60%)	53 (77.90%)	9.379	0.001
Ineffective	22 (52.40%)	15 (22.10%)		

PLR, platelet-to-lymphocyte ratio; FIGO, International Federation of Gynaecology and Obstetrics; SCC, squamous cell carcinoma.

**Table 3:** Association of PNI with clinical features of cervical cancer patients.

Variable	PNI (High)	PNI (Low)	X^2^	P
Age (year)				
<50	49 (62.00%)	12 (38.70%)	4.89	0.027
≥50	30 (38.0%)	19 (61.3%)		
FIGO stage				
I + II	40 (50.60%)	12 (38.80%)	4.01	0.26
III + IV	39 (49.40%)	19 (61.20%)		
Histological type				
SCC	72 (91.10%)	25 (80.60%)	2.35	0.125
Non-SCC	7 (8.90%)	6 (19.40%)		
Lymph node metastasis				
Negative	44 (55.70%)	13 (41.90%)	1.69	0.194
Positive	35 (44.30%)	18 (58.10%)		
Maximum tumour size (cm)				
>4 cm	48 (60.80%)	17 (54.80%)	0.32	0.57
≤4 cm	31 (39.20%)	14 (45.20%)		
Prognosis				
Effective	59 (74.70%)	14 (45.20%)	6.15	0.013
Ineffective	20 (25.30%)	17 (54.80%)		

PNI, prognostic nutritional index; FIGO, International Federation of Gynaecology and Obstetrics; SCC, squamous cell carcinoma.

**Table 4:** Univariate Cox regression analysis affecting OS.

Variable	P(OS)	HR	95%CI
Age	0.144	0.31	0.06–1.49
Lymph node metastasis	0.118	0.18	0.02–1.41
FIGO stage	0.023	4.01	1.22–13.14
Histological type	0.016	12.03	2.96–48.81
Maximum tumour size (cm)	0.158	2.91	0.69–12.26
PLR	0.043	1.01	1.00–1.03
PNI	0.032	0.89	0.79–0.99

OS, overall survival; HR, hazard ratio; CI, confidence interval; FIGO, International Federation of Gynaecology and Obstetrics; PLR, platelet-to-lymphocyte ratio; PNI, prognostic nutritional index.

**Table 5:** Univariate Cox regression analysis affecting PFS.

Variable	P(PFS)	HR	95%CI
Age	0.020	1.81	1.12–2.93
Lymph node metastasis	0.489	0.73	0.31–1.75
FIGO stage	0.151	0.80	0.59–1.08
Histological type	0.012	0.16	0.07–0.39
Maximum tumour size (cm)	0.436	1.20	0.74–1.94
PLR	0.001	2.81	1.66–4.77
PNI	0.001	0.32	0.17–0.58

PFS, progression-free survival; HR, hazard ratio; CI, confidence interval; FIGO, International Federation of Gynaecology and Obstetrics; PLR, platelet-to-lymphocyte ratio; PNI, prognostic nutritional index.

**Table 6:** Multifactorial Cox regression analysis affecting OS.

Variable	P(OS)	HR	95%CI
Age	0.165	1.40	0.39–4.55
Lymph node metastasis	0.855	0.86	0.17–4.25
FIGO stage	0.021	2.61	1.12–5.92
Histological type	0.040	10.53	3.12–35.56
Maximum tumour size (cm)	0.642	0.75	0.22–2.58
PLR	0.026	1.99	1.41–2.83
PNI	0.016	1.07	1.03–1.11

OS, overall survival; HR, hazard ratio; CI, confidence interval; FIGO, International Federation of Gynaecology and Obstetrics; PLR, platelet-to-lymphocyte ratio; PNI, prognostic nutritional index.

**Table 7:** Multifactorial Cox regression analysis affecting PFS.

Variable	P(PFS)	HR	95%CI
Age	0.042	1.77	1.03–3.04
Lymph node metastasis	0.206	1.39	0.84–2.29
FIGO stage	0.931	0.93	0.24–4.13
Histological type	0.041	3.31	1.06–10.41
Maximum tumour size (cm)	0.687	1.18	0.67–2.25
PLR	0.545	0.99	0.98–1.01
PNI	0.294	1.06	0.95–1.19

PFS, progression-free survival; HR, hazard ratio; CI, confidence interval; FIGO, International Federation of Gynaecology and Obstetrics; PLR, platelet-to-lymphocyte ratio; PNI, prognostic nutritional index.

## Data Availability

The datasets used and/or analysed during the current study are available from the corresponding author on reasonable request.

## References

[1] Buskwofie A., David-West G., Clare C.A. (2020). A Review of Cervical Cancer: Incidence and Disparities. J. Natl. Med. Assoc..

[2] Devarapalli P., Labani S., Nagarjuna N., Panchal P., Asthana S. (2018). Barriers affecting uptake of cervical cancer screening in low and middle income countries: A systematic review. Indian J. Cancer.

[3] Koh W.J., Abu-Rustum N.R., Bean S., Bradley K., Campos S.M., Cho K.R., Chon H.S., Chu C., Clark R., Cohn D. (2019). Cervical Cancer, Version 3.2019, NCCN Clinical Practice Guidelines in Oncology. J. Natl. Compr. Cancer Netw..

[4] Mahantshetty U.M. (2019). Scale-up of radiotherapy for cervical cancer. Lancet Oncol..

[5] Ilhan Z.E., Łaniewski P., Thomas N., Roe D.J., Chase D.M., Herbst-Kralovetz M.M. (2019). Deciphering the complex interplay between microbiota, HPV, inflammation and cancer through cervicovaginal metabolic profiling. EBioMedicine.

[6] Sadri Nahand J., Moghoofei M., Salmaninejad A., Bahmanpour Z., Karimzadeh M., Nasiri M., Mirzaei H.R., Pourhanifeh M.H., Bokharaei-Salim F., Mirzaei H. (2020). Pathogenic role of exosomes and microRNAs in HPV-mediated inflammation and cervical cancer: A review. Int. J. Cancer.

[7] Sun J., Mei Y., Zhu Q., Shou C., Tjhoi W.E.H., Yang W., Yu H., Zhang Q., Liu X., Yu J. (2019). Relationship of prognostic nutritional index with prognosis of gastrointestinal stromal tumors. J. Cancer.

[8] Huang Z., Fu Z., Huang W., Huang K. (2020). Prognostic value of neutrophil-to-lymphocyte ratio in sepsis: A meta-analysis. Am. J. Emerg. Med..

[9] Raungkaewmanee S., Tangjitgamol S., Manusirivithaya S., Srijaipracharoen S., Thavaramara T. (2012). Platelet to lymphocyte ratio as a prognostic factor for epithelial ovarian cancer. J. Gynecol. Oncol..

[10] Okadome K., Baba Y., Yagi T., Kiyozumi Y., Ishimoto T., Iwatsuki M., Miyamoto Y., Yoshida N., Watanabe M., Baba H. (2020). Prognostic Nutritional Index, Tumor-infiltrating Lymphocytes, and Prognosis in Patients with Esophageal Cancer. Ann. Surg..

[11] Hirahara N., Tajima Y., Fujii Y., Kaji S., Kawabata Y., Hyakudomi R., Yamamoto T. (2020). High Preoperative Prognostic Nutritional Index Is Associated with Less Postoperative Complication-Related Impairment of Long-Term Survival After Laparoscopic Gastrectomy for Gastric Cancer. J. Gastrointest Surg..

[12] Chen L., Bai P., Kong X., Huang S., Wang Z., Wang X., Fang Y., Wang J. (2021). Prognostic Nutritional Index (PNI) in Patients With Breast Cancer Treated With Neoadjuvant Chemotherapy as a Useful Prognostic Indicator. Front. Cell Dev. Biol..

[13] Pinto P.J.J., Chen M.J., Santos Neto E., Faloppa C.C., De Brot L., Guimaraes A.P.G., da Costa A., Baiocchi G. (2022). Prognostic factors in locally advanced cervical cancer with pelvic lymph node metastasis. Int. J. Gynecol. Cancer.

[14] Cupp M.A., Cariolou M., Tzoulaki I., Aune D., Evangelou E., Berlanga-Taylor A.J. (2020). Neutrophil to lymphocyte ratio and cancer prognosis: An umbrella review of systematic reviews and meta-analyses of observational studies. BMC Med..

[15] Bulut G., Ozdemir Z.N. (2022). Prognostic Significance of Neutrophil-Lymphocyte Ratio and Platelet-Lymphocyte Ratio in Metastatic Colorectal Cancer. J. Gastrointest Cancer.

[16] Wu D.M., Shi J., Liu T., Deng S.H., Han R., Xu Y. (2018). Integrated analysis reveals down-regulation of SPARCL1 is correlated with cervical cancer development and progression. Cancer Biomark.

[17] Bray F., Ferlay J., Soerjomataram I., Siegel R.L., Torre L.A., Jemal A. (2018). Global cancer statistics 2018: GLOBOCAN estimates of incidence and mortality worldwide for 36 cancers in 185 countries. CA Cancer J. Clin..

[18] Chen L., Zhang F., Sheng X.G., Zhang S.Q., Chen Y.T., Liu B.W. (2016). Peripheral platelet/lymphocyte ratio predicts lymph node metastasis and acts as a superior prognostic factor for cervical cancer when combined with neutrophil: Lymphocyte. Medicine.

[19] Bourke C.D., Berkley J.A., Prendergast A.J. (2016). Immune Dysfunction as a Cause and Consequence of Malnutrition. Trends Immunol..

[20] Francini E., Ou F.S., Lazzi S., Petrioli R., Multari A.G., Pesola G., Messuti L., Colombo E., Livellara V., Bazzurri S. (2021). The prognostic value of CD3+ tumor-infiltrating lymphocytes for stage II colon cancer according to use of adjuvant chemotherapy: A large single-institution cohort study. Transl. Oncol..

[21] Onodera T., Goseki N., Kosaki G. (1984). Prognostic nutritional index in gastrointestinal surgery of malnourished cancer patients. Nihon Geka Gakkai Zasshi.

[22] Watanabe I., Kanauchi N., Watanabe H. (2018). Preoperative prognostic nutritional index as a predictor of outcomes in elderly patients after surgery for lung cancer. Jpn. J. Clin. Oncol..

[23] Graziano V., Grassadonia A., Iezzi L., Vici P., Pizzuti L., Barba M., Quinzii A., Camplese A., Di Marino P., Peri M. (2019). Combination of peripheral neutrophil-to-lymphocyte ratio and platelet-to-lymphocyte ratio is predictive of pathological complete response after neoadjuvant chemotherapy in breast cancer patients. Breast.

[24] Ida N., Nakamura K., Saijo M., Kusumoto T., Masuyama H. (2018). Prognostic nutritional index as a predictor of survival in patients with recurrent cervical cancer. Mol. Clin. Oncol..

[25] Prabawa I.P.Y., Bhargah A., Liwang F., Tandio D.A., Tandio A.L., Lestari A.A.W., Budiana I.N.G., Manuaba I. (2019). Pretreatment Neutrophil-to-Lymphocyte ratio (NLR) and Platelet-to-Lymphocyte Ratio (PLR) as a Predictive Value of Hematological Markers in Cervical Cancer. Asian Pac. J. Cancer Prev..

[26] Xiao L., Lyu J., Liu X., Li K., Wang Y., Zhang R., Chen T., Li T. (2021). Clinical Application Value of the Prognostic Nutritional Index for Predicting Survival in Patients with Esophageal Squamous Cell Carcinoma Undergoing Chemoradiotherapy or Radiotherapy. Nutr. Cancer.

[27] Haraga J., Nakamura K., Omichi C., Nishida T., Haruma T., Kusumoto T., Seki N., Masuyama H., Katayama N., Kanazawa S. (2016). Pretreatment prognostic nutritional index is a significant predictor of prognosis in patients with cervical cancer treated with concurrent chemoradiotherapy. Mol. Clin. Oncol..

[28] Zhou M., Zhang Z., Bao S., Hou P., Yan C., Su J., Sun J. (2021). Computational recognition of lncRNA signature of tumor-infiltrating B lymphocytes with potential implications in prognosis and immunotherapy of bladder cancer. Brief Bioinform..

[29] Diem S., Schmid S., Krapf M., Flatz L., Born D., Jochum W., Templeton A.J., Früh M. (2017). Neutrophil-to-Lymphocyte ratio (NLR) and Platelet-to-Lymphocyte ratio (PLR) as prognostic markers in patients with non-small cell lung cancer (NSCLC) treated with nivolumab. Lung Cancer.

[30] Tyagi T., Jain K., Yarovinsky T.O., Chiorazzi M., Du J., Castro C., Griffin J., Korde A., Martin K.A., Takyar S.S. (2023). Platelet-derived TLT-1 promotes tumor progression by suppressing CD8+ T cells. J. Exp. Med..

[31] Rajwa P., Życzkowski M., Paradysz A., Bujak K., Bryniarski P. (2018). Evaluation of the prognostic value of LMR, PLR, NLR, and dNLR in urothelial bladder cancer patients treated with radical cystectomy. Eur. Rev. Med. Pharmacol. Sci..

[32] Song X., Chen D., Yuan M., Wang H., Wang Z. (2018). Total lymphocyte count, neutrophil-lymphocyte ratio, and platelet-lymphocyte ratio as prognostic factors in advanced non-small cell lung cancer with chemoradiotherapy. Cancer Manag. Res..

[33] Ma J.Y., Ke L.C., Liu Q. (2018). The pretreatment platelet-to-lymphocyte ratio predicts clinical outcomes in patients with cervical cancer: A meta-analysis. Medicine.

